# Enhancing the connection between the classroom and the clinical workplace: A systematic review

**DOI:** 10.1007/s40037-017-0338-0

**Published:** 2017-03-14

**Authors:** Sanne Peters, Geraldine Clarebout, Agnes Diemers, Nicolas Delvaux, An Verburgh, Bert Aertgeerts, Ann Roex

**Affiliations:** 1 Academic Center for General Practice, Department of Public Health and Primary Care, KU Leuven, University of Leuven, Leuven, Belgium; 2 School of Health Professions Education, Maastricht University, Maastricht, The Netherlands; 3 Department of General Practice, University Medical Center Groningen, Groningen, The Netherlands; 4 Education, Group Health and Wellbeing, University Colleges Leuven-Limburg UCLL, Leuven, Belgium

**Keywords:** Transfer, Systematic review, Integration

## Abstract

**Introduction:**

Although medical students are increasingly exposed to clinical experiences as part of their training, these often occur parallel with, rather than connected to, their classroom-based learning experiences. Additionally, students seem to struggle with spontaneously making the connection between these spheres of their training themselves. Therefore, this systematic review synthesized the existing evidence about educational interventions that aim to enhance the connection between learning in the classroom and its application in the workplace.

**Methods:**

Electronic databases (AMED, CINAHL, EMBASE, ERIC, Medline, RDRB, PsycINFO and WoS) were screened for quantitative and qualitative studies investigating educational interventions that referenced a connection between the classroom and workplace-based experiences within undergraduate, graduate or postgraduate medical education.

**Results:**

Three types of interventions were identified: classroom to workplace interventions, workplace to classroom interventions, and interventions involving multiple connections between the two settings. Most interventions involved a tool (e. g. video, flow chart) or a specific process (e. g. linking patient cases with classroom-based learning content, reflecting on differences between what was learned and how it works in practice) which aimed to enhance the connection between the two settings.

**Discussion:**

Small-scale interventions can bring classroom learning and workplace practice into closer alignment. Such interventions appear to be the necessary accompaniments to curricular structures, helping bridge the gap between classroom learning and workplace experience. This paper documents examples that may serve to assist medical educators in connecting the classroom and the workplace.

**Electronic supplementary material:**

The online version of this article (doi: 10.1007/s40037-017-0338-0) contains supplementary material, which is available to authorized users.

## What this paper adds

Integrated curricula, where workplace-based experiences and classroom-based learning are woven together to the advantage of students’ learning, have become vogue in medical education. However, often these twin components solely parallel each other, rather than connect. This literature review focuses on educational interventions that aim to strengthen the connection between workplace and classroom in order to support students’ application of acquired competences. Interventions such as those documented in this paper may serve as examples to assist medical educators. This paper also offers insight into the scope and methodological rigour of the work that has been conducted in this field to date.

## Introduction

With the importance of the transfer of learning now widely acknowledged in the education sector, the ability to apply and refine learned competences across contexts different from those in which the initial learning took place has become a major goal of education [1, 2]. To achieve this goal within medical education, institutions have tried to enrich classroom-based learning with (early) clinical experience. Despite the increasing popularity of curricula that add these clinical experiences to classroom-based learning, so-called ‘vertically integrated curricula’, students still struggle with the transfer of learning [3]. The problem is that these workplace experiences are often conducted in an isolated way, alongside classroom-based learning, rather than connected to the learning experience [4]. This connection is important as it allows students to learn how, when, where and why to apply competences gained in the classroom into practice [5]. Furthermore, it seems that students are not frequently able to spontaneously make this connection between the two settings [6, 7], and this may impede the transfer of learning. Hence, there is a need to make the connection between classroom learning and its application in the workplace more explicit [8]. The challenge is in optimizing the connection between both settings in order to improve the transfer of learning.

This transfer of learning appears to be a complex and dynamic process [9–12], due to the context-dependent character of learning and the many influencing factors [13]. Transfer of learning is an ongoing process rather than a range of discrete acquisition events [14] and it can be perceived as a cyclical process consisting of the following six interrelated stages: 1) selection of potentially relevant competences from familiar context(s); 2) understanding the new situation; 3) recognizing what is needed; 4) transforming prior competences to fit the new context; 5) integrating them with other competences in order to act in the new situation [15] and 6) learning from their application [16, 17]. The next cycle commences when a new situation is encountered, building on the competences developed in previous cycles.

Three major groups of variables influencing the transfer of learning were identified in recently published reviews [11, 12], namely:

1. The training design: this relates to the learning environment in which competences are acquired. Within medical education this is referred to as the medical curriculum (e. g. content relevance, opportunity to practice and feedback).
2. Learner characteristics: this involves contributing factors related to the individual student (e. g. prior knowledge, self-efficacy, motivation to transfer).
3. The work environment: this refers to the job context where the learned competences are applied. Within medical education this is called the clinical workplace (e. g. opportunity to perform, supervisory support).

Medical students experience difficulties transferring what they have learned in the classroom (where students lay theoretical and practical foundations) to the clinical workplace (where students practice with real patients) [6, 7, 9]. More so, students are often unable to make the connection between the two settings [6, 7]. This even appears to be the case within medical curricula that combine classroom-based learning (e. g. lectures, workshops, online learning, simulation-based training, self-study) with workplace-based learning (e. g. early clinical exposure, internship, clerkship, residency).

This systematic review synthesized the existing literature about educational interventions that aim to enhance the connection between the classroom and the clinical workplace. The urge to answer questions about why, when, where and how educational interventions work guided this systematic review [18, 19]. Those systematic reviews which solely fo﻿cus on ‘whether’ an educational intervention was effective have been criticized for their limited relevance within medical education [18]. The weakness of many systematic reviews in medical education was their tendency to use statistical methods, such as a meta-analysis, which are commonly used to answer clinical questions [18]. Given that educational systematic reviews often encounter a lot of heterogeneity, the results of such analyses are difficult to interpret [18]. Therefore, both quantitative as well as qualitative sources of evidence were considered in this review [18–20]. Moreover, the literature synthesis aimed to get insight into the scope and rigour of the research that has been done in the field.

## Methods

This systematic review was conducted in accordance with the guidelines of Best Evidence in Medical Education [21]. The reporting was based on the Preferred Reporting Items for Systematic Reviews and Meta-Analyses (PRISMA) guidance for systematic reviews [22].

### Search strategy

The following electronic databases were searched for primary studies between January 2004 and October 2014: AMED, CINAHL, EMBASE, ERIC, Medline, RDRB, PsycINFO and WoS. Three main search terms were identified: medical education, workplace learning and transfer of learning (see online Supplementary Data for a list of all the search terms). The search terms were selected via a consultative process, taking into account the inclusion criteria, an exploratory search of the relevant literature and browsing the MEDLINE Thesaurus of subject indexing terms. The search terms, subject headings as well as free text words were combined in the search strategy for each database. Scoping searches were conducted to refine the search strategy, a research librarian was consulted for advice and experts in the field were contacted to make sure that relevant articles were not omitted. Moreover, a search of the reference lists of the included articles and of topic-related systematic reviews identified possible additional relevant studies.

### Study selection

After elimination of duplicates, the studies were selected in two phases. First, relevant articles were selected independently by two reviewers based on title and abstract. One reviewer (SP) conducted the initial screening of all titles and abstracts, which were then divided between three reviewers (AV, AD and ND) for a second screening. The full article was retrieved when the reviewer was unsure of its relevance. Disagreements about inclusion or exclusion were resolved through discussion. Articles were included based on the following inclusion criteria:

- Peer-reviewed studies focusing on level of undergraduate, graduate or postgraduate medical education,
- Studies containing educational interventions of any duration, medium or format that referenced a connection between the classroom and the workplace,
- All types of study design and both quantitative and qualitative primary research studies, whatever the outcome measures.

The exclusion criteria were:

- Articles about physicians and their continuous professional development,
- Studies about other health professions (e. g. nursing),
- Studies concerning interventions in simulation settings with no connection to workplace learning,
- Articles which were not available in the English language.

In the second phase, the full texts of the selected studies were independently screened for eligibility by two reviewers (SP and AR or GC). Agreement was reached via discussion. If necessary, a third reviewer was consulted.

### Quality appraisal

The full text of each selected study was retrieved and two reviewers independently assessed the methodological quality (SP and AR or GC). Disagreements were resolved through discussion. To assess the quality of included studies, a series of 11 quality ‘indicators’ were used ([23]; Table 1). These related to the appropriateness of the study design, conduct, results analysis and conclusions. Higher quality studies were considered to be those that met a minimum of seven of these 11 indicators [23].

**Table 1:** Quality indicators [23]

A.	Research question: Is the research question(s) or hypothesis clearly stated?
B.	Study subjects: Is the subject group appropriate for the study being carried out (number, characteristics, selection, and homogeneity)?
C.	‘Data’ collection methods: Are the methods used (qualitative or quantitative) reliable and
D.	Valid for the research question and context?
E.	Completeness of ‘data’: Have subjects dropped out? Is the attrition rate less than 50%? For questionnaire-based studies, is the response rate acceptable (60% or above)?
F.	Control for confounding: Have multiple factors/variables been removed or accounted for where possible?
G.	Analysis of results: Are the statistical or other methods of results analysis used appropriate?
H.	Conclusions: Is it clear that the data justify the conclusions drawn?
I.	Reproducibility: Could the study be repeated by other researchers?
J.	Prospective: Does the study look forwards in time (prospective) rather than backwards (retrospective)?
K.	Ethical issues: Were all relevant ethical issues addressed?
L.	Triangulation: Were results supported by data from more than one source?

### Data extraction and analysis

A data extraction form was developed based on the Best Evidence in Medical Education (BEME) coding sheet [23]. This form was piloted on a few included studies and iteratively refined until the form adequately captured all the extractable data that were relevant for the research question, such as the number of participants and study design. The data extraction form evolved, as shown in the online Supplementary Data. The studies were also compared with the six interrelated stages of the cyclical transfer process [15–17]. The outcome measures of the studies were classified using Miller’s pyramid, which is a framework for mapping assessment methods of clinical competence [8]. Tests assessing knowledge are situated at the lowest level of the pyramid (‘knows’ level), followed by tests which map out the application of knowledge (‘knows how’ level), assessment methods for demonstration of clinical skills (‘shows how’ level) and assessment of daily patient care (‘does’ level). Given that this classification strategy is widely used within medical education, accommodating both classroom-based and workplace-based assessment methods within its hierarchy, it was deemed suitable for grouping the outcome measures. Data were extracted by one reviewer (SP) and independently checked by a second reviewer (AR or GC). Differences in opinion were resolved through discussion. In the case of important missing data, e. g. detailed description of the intervention, attempts were made to contact the authors of the original paper.

## Results

### Search results

**Fig. 1:**
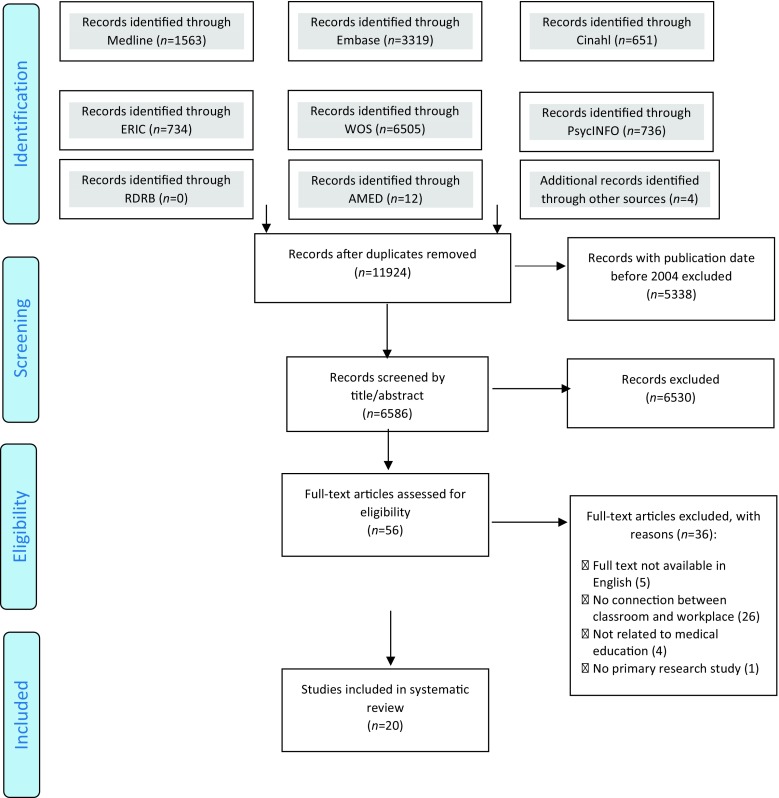
Flow diagram of the study selection process according to PRISMA (Preferred reporting items for systematic reviews and meta-analyses) criteria [22]

The search strategy identified 11,924 papers once duplicates were removed (Fig. 1). Due to the large number of search results generated by the search strategy, records with publication date before 2004 were excluded (*n* = 5338). The number of publications about integrated curricula and, therefore, the relevance of interventions that connect the classroom with the workplace has grown significantly over the last decade in medical education [3]. Moreover, given that teaching methods in medical education are evolving, the researchers consider that the last ten years are the most relevant.

Of the screened abstracts (*n* = 6586), 6530 papers were excluded mainly because they did not contain a connection between classroom-based and workplace-based learning. Moreover, the large number of exclusions was attributable to the difficulty in finding clear search terms for studies that focused on a connection. This resulted in the need to consult both literature on classroom-based learning and literature on workplace-based learning. In turn, this approach yielded many irrelevant abstracts. Of the 56 papers that were assessed for eligibility, 36 were excluded for several reasons: full texts not available in English (*n* = 5); no connection between classroom and workplace (*n* = 26); not related to medical education (*n* = 4); no primary research study (*n* = 1). Finally, 20 papers were included for the data extraction and analysis.

### Description of the included studies

#### Participants

The educational level of the participants in the included studies varied (see online Supplementary Data). Most participants were in the third year of the medical undergraduate course. The majority (*n* = 9) of the studies were from the USA. Other participants came from Bahrain, the UK, Canada, the Netherlands and Denmark. The setting of the study was described briefly in most cases. However, this was often limited to the name of the university or medical school. Only one study provided a description of the learning environment and curriculum in which the intervention took place. This study was situated in a problem-based student-centred undergraduate medical curriculum [24].

#### Intervention – description

Three types of interventions were identified: Classroom to workplace interventions, workplace to classroom interventions and interventions involving multiple connections between classroom and workplace. See the online Supplementary Data for examples of each type of interventions. The stages of the transfer process that were present in each intervention are indicated in this supplement. It was not always clear whether the sixth stage was reached. This uncertainty was indicated by ‘6?’.

Most of the interventions (*n* = 15) contained an educational tool, developed by the medical teacher, which aimed to stimulate the transfer of learning. These tools included a video demonstration illustrating a medical performance [25, 26], a learning task or assignment [27–31], a portfolio [32, 33], an email containing key messages of the classroom-based session [34, 35], classroom-based learning materials in the form of either a text or a practical protocol or flow chart [36, 37], an unannounced simulation patient [38], a logbook [39] and an observation form [40]. These tools appeared to have three functions, namely to deliberately practice a specific competence at the workplace (*n* = 10), to offer just-in-time information at the moment that the student needs it (*n* = 5) and to refresh prior learned competences (*n* = 5). Some of the educational tools focused on more than one of these three functions.

Fifteen out of the 20 studies involved processes of supervision that aimed to enhance the transfer of learning, namely formulating learning goals with students [31, 36], reviewing prior learned competences [32, 33, 36], linking patient cases with classroom-based learning content [24, 33, 41, 51], modelling the application of the competences [42], offering opportunity to practice with real patients (*n* = 9), providing feedback to the students (*n* = 6) and facilitating reflection after clinical work (*n* = 5). These supervision processes were facilitated by a medical doctor, either on a one-on-one basis or in a small group of students. In one case the supervision was guided by a resident [39].

#### Intervention – duration/frequency

The interventions varied in duration and frequency. Some interventions took place over a short period of time, e. g. a 3-minute video viewed once [26]. Other interventions ran over a longer period, e. g. 8 practice days over 18 months [30]. The frequency of the interventions varied from once, e. g. one encounter with a simulated patient [38], to several times, e. g. once a week [34]. In the online Supplementary Data the description of each element of the intervention (e. g. skills course, video instruction, feedback session, …) is itemized using roman numerals (e. g. i, ii, iii, …) and recorded alongside its duration and/or frequency (e. g. i. 70 min, ii. 5 min, iii. 60 min).

#### Study designs, outcome measures and methodological quality

There was a wide variety of study designs: evaluation studies (*n* = 7), controlled trials (CT) (*n* = 4), randomized controlled trial (RCT) designs (*n* = 3), uncontrolled pre- and post-designs (*n* = 3), post-intervention study (*n* = 1), qualitative study (*n* = 1) and pilot cohort study (*n* = 1). There was also a broad range of outcome measures. Eleven interventions employed outcome measures that met one of the levels on Miller’s pyramid, three on the ‘knows’ level, two on the ‘knows how’ level, four on the ‘shows how’ level and two on the ‘does’ level of pyramid [8]. In nine of the included studies, the interventions were not assessed using outcome measures that met any level of Miller’s pyramid. In these nine cases, the results were generated exclusively from students’ perceptions recorded in interviews, focus groups or questionnaires. Six studies contained a component of students’ self-assessment and six studies were only based upon medical teachers’ and/or workplace supervisors’ assessment. Most outcomes were measured immediately after the intervention. In four studies the outcomes were measured over a longer period of time.

An assessment of the methodological quality of each included study, based on the 11 quality indicators (QI), is shown in the online Supplementary Data. The table records each study’s QI score out of maximum 11, (e. g. QI Score = 9/11), as well as the specific quality indicators that were met in the study (e. g. QI met: A, B, C, D, E, F, G, H and K). There was only one study [4] that was excluded because of poor methodological quality.

#### Study outcomes

Due to the variety of study designs and outcome measures, there was a lot of heterogeneity in study outcomes (online Supplementary Data).

With regard to the type of interventions from the classroom to the workplace, the study which focused on a urethral catheterization skills course with practice opportunities on mannequins (classroom) had the highest quality, taking into account the QI score, the study design and the level of Miller’s pyramid (online Supplementary Data) [25]. Students watched an instructive video which aimed at refreshing previously learned competences both after their classroom-based session and immediately before performing an urethral catheterization with a real patient (workplace). The outcomes of this RCT were measured after the urethral catheterization course and after the performance with a real patient. No differences between the intervention and the control group were found [25]. This study was situated on the ‘does’ level of Miller’s pyramid and had a QI score of 9.

Concerning the type of interventions from the workplace to the classroom, the study by Davis et al. had the highest quality and indicated the largest effect size. While caring for real patients (workplace), the intervention group watched a 3-minute video demonstrating proper chest tube placement, to remind them of their prior knowledge and skills (classroom) [26]. This controlled trial showed that the intervention group performed significantly better than the control group (11.1 ± 3.09 versus 7.2 ± 3.6, *p* < 0.001). The Cohen’s D value for effect size was calculated at 12 [26], which exceeds Cohen’s convention for large effect. This study was situated on the ‘shows how’ level of Miller’s pyramid and had a QI score of 10.

For the interventions involving multiple connections between classroom and workplace, the study of Kerfoot et al. had the highest quality [35]. The participants followed a web-based teaching program on urology (classroom), followed by a urology rotation (workplace). After completing the rotation, participants received weekly emails to refresh their acquired competences from the web-based teaching program (classroom). This RCT used pre- and post-knowledge tests, an end-of-year knowledge test and self-reported email utilization patterns as outcome measures [35]. The intervention group scored higher on the tests than the control group. This study was positioned on the ‘knows’ level of Miller’s pyramid and had a QI score of 11.

Generally, in the studies where an intervention group was compared with a control group (*n* = 7), the intervention group scored the same as the control group (*n* = 1) or better (*n* = 3) [26, 35, 38]. The intervention group never scored worse than the control group. In some studies with multiple outcome measures (*n* = 4), the intervention group scored the same as the controls for some outcome measures and better on some other outcome measures. These multiple outcome measures and the corresponding outcomes were indicated in the online Supplementary Data using an alphabetic sequence (e. g. a, b, c, …).

## Discussion

This systematic review synthesized interventions that aimed to enhance the connection between classroom and workplace. The topic of integrated curricula is popular and there is a lot of literature about learning in the classroom and the workplace. However, there were only 20 studies that met the strict inclusion criteria of this systematic review. The review showed that a wide variety of interventions exist with rather positive results. Nevertheless, some of them need to be interpreted carefully due to methodological issues. This literature synthesis gives an insight into the scope and rigour of the research that has been done in this area. Three types of educational interventions were identified: classroom to the workplace interventions, workplace to the classroom interventions, and interventions involving multiple connections between classroom and workplace. A range of educational tools and supervising processes that aimed to enhance the connection between the two settings were identified. Three studies will be highlighted. The most successful intervention entailed a video demonstration to refresh classroom-acquired knowledge and skills. It was identified as the most successful, based on the following criteria: study outcomes were positive (intervention group > control group), appropriate study design, high QI score and ‘shows how’ level of Miller’s pyramid [26]. Yet, this intervention only comprised the first stage of the cyclical process. The study with the highest methodological rigour comprised a QI score of 11, aimed five stages of the cyclical process, a RCT design but outcomes were only assessed at the ‘knows’ level of Miller’s pyramid [35]. The intervention involved a web-based training programme followed by weekly educational emails while caring for real patients. The study outcomes were positive [35]. The most promising study was the one by Van Weel-Baumgarten et al., even though the study outcomes were less clear [43]. Participants followed sessions about communication, practice with simulated patients and received feedback. After practising with real patients during the clerkship, they reflected on their communication in small groups and individual counselling sessions took place. This intervention seems promising because 5 (or 6?) stages of the cyclical transfer process were covered in this intervention. However, only perceptions of the course were taken into account as outcome measure.

The three types of interventions reported in this systematic review each reflected elements of the cyclical transfer of learning process. First, the interventions that dealt with the classroom to workplace transition seemed to prepare students mainly for the first five stages of the cyclical transfer of learning process, namely selection of potentially relevant competences from familiar context(s); understanding the new situation; recognising what is needed; transforming prior competences to fit the new context; and integrating prior competences with other competences in order to act in the new situation [15].

Second, those interventions targeting the transfer of learning from the workplace to the classroom were staged at the workplace and designed to support students in making connections back to classroom-based learning. These interventions mainly seemed to concern one particular stage of the cyclical process, namely aiding students in the selection of potentially relevant competences from familiar context(s) [15]. This type of intervention seemed to emphasize the refreshment of prior learned competences.

Third, the interventions with multiple connections between the classroom and the workplace seemed to involve all the above-mentioned stages of the cyclical process. Most studies in this systematic review commented on the workplace supervisor’s task to provide feedback and facilitate self and group reflection. This important role of the supervisors was also identified in previous research [8]. The place of feedback and reflection within the interventions might be linked to the final stage in the cyclical transfer of learning process, namely learning from application [16, 17]. Yet, this was not clear because no study made explicit reference to the specific type of feedback or reflection they used. There are many different types of *feedback*, e. g. corrective and cognitive feedback [44], and *reflection*, e. g. reflection-in-action, reflection-on-action and reflection-on-competency [45]. Depending on which type of feedback or reflection was used in the study, the final stage of the cyclical process might or might not be engaged. Moreover, it was not mentioned whether the feedback or reflection referred to what was learned in the classroom and, therefore, if an explicit connection was made between the two settings.

The majority of the interventions in this systematic review also contained a tool to stimulate transfer of learning, e. g. a video demonstration, a flow chart, a learning task or a logbook. These educational tools were used in both the classroom and the workplace setting. This is in alignment with previous research that refers to *transfer tools* or *boundary tools* as instruments which cannot be allocated to one setting but function across the classroom and workplace in order to enhance the connection between the two [46]. These tools stimulate boundary crossing and collaboration between the classroom and the workplace. They have been shown to be useful in promoting transfer of learning [46, 47].

This systematic review identified a number of weaknesses in the methodological approaches of the interventional studies, many of which are regarded as common features of research relating to medical education [48]. Firstly, many of the studies (*n* = 6) evaluating the impact of the intervention used students’ self-assessment and often were measured through locally developed instruments without reports of validity and reliability. Previous research suggested that self-reported perceived knowledge, skills or behaviours are loosely connected to their objective measurements [49]. Secondly, only two studies assessed the impact of interventions upon the students’ performance at the workplace. Previous research indicated the importance of measuring the students’ performance in the context in which it takes place [8, 10, 14]. Thirdly, only four of the studies included in this systematic review measured outcomes after an extended period of time. While longer term transfer is known to be difficult to measure and not frequently documented [10], research indicates that results measured by a post-test taken shortly after an educational intervention might not be maintained over a longer period of time [35]. Measuring longer term learning is an important outcome variable by which educational interventions should be evaluated [35]. Finally, it is important to understand the mechanisms of change underlying the interventions in order to collate information, which can be difficult to establish when contextual information is not provided in published papers [50]. The studies reported in this systematic review did not include rich descriptions of the intervention and only one study clarified the context in which it was set. This is in line with previous research indicating that the quality of descriptions of interventions in medical education publications often remains poor [48].

### Limitations

A limitation is that this systematic review did not include grey literature. Consequently, relevant interventions might have been unreported. However, one of our selection criteria was peer-reviewed articles to guarantee methodological quality. Additionally, the search strategy was comprehensive and developed with the help of a librarian, using the major databases for medical and educational research and following guidelines for the conducting and reporting of systematic reviews.

There was heterogeneity of the studies on a number of levels, e. g. variety of study designs, types of interventions and outcome measures, and the complexity of the interventions. The challenges arising from heterogeneity have been recognized in previous research with regards to systematic reviews of educational studies [50].

Despite these limitations, conducting a systematic review in this area was still relevant. If medical education is to be truly evidence-based, even those aspects of this discipline as yet untested by rigorous methodological approaches still require the systematic collection and synthesis of all available evidence [21]. Although studies with less rigorous methodological approaches feature prominently in this systematic review, their prevalence merely reflects the emergent nature of this field of research which has only recently come under the scrutiny of academic inquiry. While the studies reported on in this paper contain ideas that could give inspiration for educational practice and further research, this systematic review attests to the need for more high-quality research in this area of medical education.

It is recognized that secondary research ought to extend beyond evaluating the effectiveness of interventions into richer descriptions about why, when, where and how educational interventions work [19]. However, before this can occur, primary research needs to incorporate the relevant details that give shape and context to their findings, which in turn will provide the firm ground upon which secondary research can construct a synthesis of ‘clarification research’ [20]. This will allow secondary research to focus on why, for whom and in which circumstances educational interventions are effective [20].

### Implications for practice and future research

Well-placed and small-scale interventions such as these in the included studies appear to be necessary accompaniments to curricular structures that parallel workplace experiences with classroom learning [3]. With the increasing focus on integrated curricula which simply add workplace-based experiences to classroom-based learning [4], there is a need to make the connection between learning in the classroom and its application in the workplace more explicit [8]. This systematic review showed practical examples of how to manage this within medical education. Moreover, the inclusion of the QI score, level of Miller’s pyramid and the stages of the cyclical process, allow medical educators to easily verify the relevance and methodological quality of each intervention in this systematic review. Generally, the outcomes of the included studies are hopeful but the methodological aspects are quite diverse. Taking into account the quality of the study, the intervention that was most successful contained a 3-minute video demonstration to refresh classroom-acquired knowledge and skills [26]. The studies that did not yield highly positive results mainly contained limited outcome measures (e. g. solely perceptions of the course). Given that only 20 studies met the inclusion criteria of this systematic review, it is possible that many interventions connecting the classroom with the workplace exist but simply have not been reported. This field of medical education would benefit from more primary research, specifically studies containing detailed descriptions of the interventions, as well as descriptions of the contexts in which they are taking place. It is also recommended that future research measures the outcomes of the intervention on the ‘does’ level of Miller’s pyramid [8], over an extended period of time after the intervention [35], and compares results with a control group. This systematic review emphasizes that several actors play a role in the transfer of learning. These actors might have different conceptions about the transfer, alongside the ones that were identified in educational research [47], but these are not specified yet. Future research could investigate these conceptions and their impact on the transfer of learning. Moreover, future research needs to establish whether feedback and reflection, which explicitly connects what was learned in the classroom with workplace experiences, strengthens the connection between the two settings and, therefore, enhances the transfer of learning.

## Conclusion

This systematic review showed that the use of well-placed and small-scale approaches, e. g. by using transfer tools and/or supervising processes, might bring classroom learning and workplace practice into closer alignment. The studies included seem to have mainly targeted the first five stages of the transfer of learning process, which is known to be characterized by a cycle of six interrelated stages.

This review presents practical examples of how to strengthen the connection between the two settings, which is relevant for medical educators. It also adds to the current literature by offering insight into the scope and methodological rigour of the work that has been done in this field. In order for this emerging area of medical education to fully mature to the point at which they can be regarded as truly evidence-based, the systematic collection and synthesis of all available evidence is required.

## Caption Electronic Supplementary Material

Supplementary file 1: Search terms according to each database

Supplementary file 2: Characteristics of included studies: interventions
