# Supplementary material for: Enhancing the connection between the classroom and the clinical workplace: A systematic review
Source: Perspect Med Educ. 2017 Mar 14;6(3):148–57. doi: 10.1007/s40037-017-0338-0 (PMC5466563; doi:10.1007/s40037-017-0338-0)
Supplement: Supplementary file 1 — Search terms according to each database [file 40037_2017_338_MOESM1_ESM.docx]

**Supplementary file 1: Search terms according to each database**

| **Database** | **Population** | **Intervention** | **Outcome** |
| --- | --- | --- | --- |
| Medline | Medical education.mp. OR Education, Medical/ OR undergraduate medical education.mp. OR Education, Medical, Undergraduate/ OR graduate medical education.mp. OR Education, Medical, Graduate/ OR postgraduate medical education.mp. OR clinical education.mp. OR Students, Medical/ OR medical student*.mp. OR trainee*.mp. OR clerk*.mp. OR resident*.mp. | School* of medicine.mp. OR medical school*.mp. OR Schools, Medical/ OR medical course*.mp.  OR medical curriculum.mp. OR classroom.mp. OR training setting.mp. OR clinical phase.mp. OR Preceptorship/ OR preceptorship*.mp. OR "Internship and Residency"/ OR internship*.mp. OR residenc*.mp. OR clinical clerkship.mp. OR Clinical Clerkship/ OR clinical practice*.mp. OR clinical placement*.mp. OR clinical rotation*.mp. OR clinical experience.mp. OR clinical learning.mp. OR clinical setting*.mp. OR ambulatory care setting*.mp. OR practice based learning.mp. OR experience based learning.mp. OR work experience.mp. OR workplace learning.mp. OR Mentors/ OR mentor*.mp. | learning transfer.mp. OR "Transfer (Psychology)"/ OR training transfer.mp. OR (transfer adj5 learning).mp. OR (transfer adj5 training).mp. OR (transfer adj5 knowledge).mp. OR (transfer adj5 skill*).mp. OR (theory adj5 practice).mp. OR (appl* adj5 knowledge).mp. OR (appl* adj5 skill*).mp. OR (practicing adj5 skill*).mp. OR (practising adj5 skill*).mp. OR (integrat* adj5 knowledge).mp. OR (integrat* adj5 skill*).mp. OR transition.mp. OR cross boundary learning.mp. |
| Embase | resident* OR clerk* OR trainee* OR medical NEAR/2 student* OR 'clinical education'/de OR 'clinical education' OR 'postgraduate medical education'/de OR 'postgraduate medical education' OR 'medical education'/de OR 'medical education' | mentor* OR 'workplace learning' OR 'work experience'/de OR 'work experience' OR 'experience based learning' OR 'practice based learning' OR 'ambulatory care setting' OR clinical AND setting* OR 'clinical learning' OR 'clinical experience' OR clinical NEXT/1 rotation* OR 'clinical placement' OR 'clinical practice'/de OR 'clinical practice' OR 'clinical clerkship' OR 'residency education'/de OR 'residency education' OR residenc* OR internship* OR preceptorship* OR clinical NEXT/1 phase OR training NEXT/1 setting OR classroom OR medical NEXT/1 curriculum OR medical NEXT/1 course* OR 'medical school'/de OR 'medical school' OR 'school of medicine' | 'cross boundary learning' OR transition OR integrat* NEAR/5 skill* OR integrat* NEAR/5 knowledge OR practising NEAR/5 skill* OR practicing NEAR/5 skill* OR appl* NEAR/5 skill* OR appl* NEAR/5 knowledge OR theory NEAR/5 practice OR transfer NEAR/5 skill* OR transfer NEAR/5 knowledge OR transfer NEAR/5 training OR transfer NEAR/5 learning OR 'training transfer' OR 'learning transfer' |
| Cinahl | "medical education" OR (MH "Education, Medical") OR "clinical education" OR (MH "Education, Clinical") OR (MH "Students, Medical") OR "medical student*" OR trainee* OR "clerk*" OR "resident*" OR (MH "Interns and Residents") | "medical school*" OR "school* of medicine" OR (MH "Schools, Medical") OR "medical curriculum" OR "medical course*" OR "training setting" OR "classroom" OR (MH "Learning Environment, Clinical") OR "clinical phase" OR "clinical rotation*" OR (MH "Student Placement") OR "clinical placement" OR "clinical practice" OR "clerkship" OR "clinical clerkship" OR "residenc*" OR (MH "Internship and Residency") OR "internship*" OR "preceptorship*" OR (MH "Preceptorship") OR "workplace learning" OR (MH "Job Experience") OR (MH "Work Experiences") OR "work experience" OR "experience based learning" OR "practice based learning" OR "ambulatory care setting*" OR "clinical setting" OR (MH "Clinical Supervision") OR "clinical learning" OR MH "Fieldwork") OR "clinical experience" OR (MH "Mentorship") OR "mentor*" | "learning transfer" OR (MH "Transfer (Psychology)") OR "training transfer" OR transfer N2 training OR "transfer N2 learning" OR transfer N5 skill*  OR transfer N5 knowledge OR (MH "Professional Practice, Theory-Based") OR (MH "Theory-Practice Relationship") OR "theory practice" OR appl* N5 skill* OR appl* N5 knowledge OR practising N5 skill* OR practicing N5 skill* OR integrat* N5 skill* OR integrat* N5 knowledge OR "transition" OR "cross boundary learning" |
| ERIC | Medical education.mp. OR exp Medical Education/ OR undergraduate medical education.mp. OR graduate medical education.mp. OR exp Graduate Medical Education/ OR postgraduate medical education.mp. OR clinical education.mp. OR exp Medical Students/ OR medical student*.mp. OR exp Trainees/ OR trainee*.mp. OR clerk*.mp. OR resident*.mp. | School* of medicine.mp. OR medical school*.mp. OR exp Medical Schools/ OR medical course*.mp. OR medical curriculum.mp. OR exp Virtual Classrooms/ OR exp Classrooms/ OR classroom.mp. OR training setting.mp. OR clinical phase.mp. OR preceptorship*.mp. OR exp Experiential Learning/ OR exp Internship Programs/ OR internship*.mp. OR residency.mp. OR clinical clerkship.mp. OR clinical practice*.mp. OR clinical placement*.mp. OR clinical rotation*.mp. OR clinical experience.mp. OR exp Clinical Experience/ OR clinical learning.mp. OR clinical setting*.mp. OR ambulatory care setting*.mp. OR practice based learning.mp. OR exp Education Work Relationship/ OR experience based learning.mp. OR work experience.mp. OR exp Work Experience/ OR exp Work Experience Programs/ OR workplace learning.mp. OR exp Workplace Learning/ OR exp Mentors/ OR mentor*.mp. | exp "Transfer of Training"/ OR learning transfer.mp. OR exp "Retention (Psychology)"/ OR training transfer.mp. OR (transfer adj5 learning).mp. OR (transfer adj5 training).mp. OR (transfer adj5 knowledge).mp. OR (transfer adj5 skill*).mp. OR (theory adj5 practice).mp. OR exp Theory Practice Relationship/ OR (appl* adj5 knowledge).mp. OR (appl* adj5 skill*).mp. OR (practicing adj5 skill*).mp. OR (practising adj5 skill*).mp. OR (integrat* adj5 knowledge).mp. OR (integrat* adj5 skill*).mp. OR exp Integrated Curriculum/ OR transition.mp. OR cross boundary learning.mp. |
| WOS | Medical education OR clinical education OR medical student* OR trainee* OR Clerk* OR resident* | School* of medicine OR medical school* OR medical course* OR medical curriculum OR classroom OR training setting OR preceptorship* OR internship* OR residenc* OR clinical clerkship OR clinical placement OR clinical rotation OR clinical experience OR clinical learning OR clinical setting OR ambulatory care setting OR practice based learning OR experience based learning OR work experience OR workplace learning OR mentor* | Learning transfer OR transfer of learning OR training transfer OR transfer of training OR transfer of knowledge OR transfer of skill* OR theory and practice OR appl* of knowledge OR appl* of skill* OR practicing skill* OR practising skill* OR integrat* knowledge OR integrat* skill* |
| PsycINFO | Medical education OR clinical education OR medical student* OR trainee* OR Clerk* OR resident* | School* of medicine OR medical school* OR medical course* OR medical curriculum OR classroom OR training setting OR clinical phase OR preceptorship* OR internship* OR residenc* OR clinical clerkship OR clinical placement OR clinical rotation OR clinical experience OR clinical learning OR clinical setting OR ambulatory care setting OR practice based learning OR experience based learning OR work experience OR workplace learning OR mentor* | Learning transfer OR transfer adj5 learning OR training transfer OR transfer adj5 training OR transfer adj5 knowledge OR transfer adj5 skill* OR theory adj5 practice OR appl* adj5 knowledge OR appl* adj5 skill* OR practicing adj5 skill* OR practising adj5 skill* OR integrat* adj5 knowledge OR integrat* adj5 skill* OR transition OR cross boundary learning |
| RDRB | Medical education OR clinical education OR medical student* OR trainee* OR Clerk* OR resident* | School* of medicine OR medical school* OR medical course* OR medical curriculum OR classroom OR training setting OR clinical phase OR preceptorship* OR internship* OR residenc* OR clinical clerkship OR clinical practice OR clinical placement OR clinical rotation OR clinical experience OR clinical learning OR clinical setting OR ambulatory care setting OR practice based learning OR experience based learning OR work experience OR workplace learning OR mentor* | Learning transfer OR transfer of learning OR training transfer OR transfer of training OR transfer of knowledge OR transfer of skill* OR theory and practice OR appl* of knowledge OR appl* of skill* OR practicing skill* OR practising skill* OR integrat* knowledge OR integrat* skill* OR transition OR cross boundary learning |
| AMED | Medical education OR clinical education OR medical student* OR trainee* OR Clerk* OR resident* | School* of medicine OR medical school* OR medical course* OR medical curriculum OR classroom OR training setting OR clinical phase OR preceptorship* OR internship* OR residenc* OR clinical clerkship OR clinical practice OR clinical placement OR clinical rotation OR clinical experience OR clinical learning OR clinical setting OR ambulatory care setting OR practice based learning OR experience based learning OR work experience OR workplace learning OR mentor* | Learning transfer OR transfer adj5 learning OR training transfer OR transfer adj5 training OR transfer adj5 knowledge OR transfer adj5 skill* OR theory adj5 practice OR appl* adj5 knowledge OR appl* adj5 skill* OR practicing adj5 skill* OR practising adj5 skill* OR integrat* adj5 knowledge OR integrat* adj5 skill* OR transition OR cross boundary learning |
