# Supplementary material for: Enhancing the connection between the classroom and the clinical workplace: A systematic review
Source: Perspect Med Educ. 2017 Mar 14;6(3):148–57. doi: 10.1007/s40037-017-0338-0 (PMC5466563; doi:10.1007/s40037-017-0338-0)
Supplement: Supplementary file 2 — Characteristics of included studies: interventions [file 40037_2017_338_MOESM2_ESM.docx]

| **Supplementary file 2:** Characteristics of included studies: interventions | | | | | | |
| --- | --- | --- | --- | --- | --- | --- |
| **Reference** | **Participants** | **Intervention** | | **Study designs/ outcome measures** | **QI score** | **Study outcomes** |
|  |  | **Description** | **Duration / frequency** |  |  |  |
|  |  | ***Classroom to workplace interventions:*** |  |  |  |  |
| Todsen et al. (2013)  [1] | 28 fourth year students (pilot study) + 76 third year students (main study – 33 IG and 31 CG) | 1. C: Urethral catheterization (UC) skills course with practice on a mannequin. 2. W: Video instruction, illustrating a physician performing UC on a patient with a voice-over explaining the difficult steps, immediately before performing a UC on a real patient.  - CG: Participation in UC course but no video instruction.   *🡪 Stages of cyclical process present: 1, 2, 3, 4 and 5* | 1. 70 minutes 2. 5 minutes (once only) | *RCT*   1. Post-test (immediately after course): scenario with a mannequin. 2. Transfer test (1 or 6 weeks after course and immediately after video): Performing UC on a patient^E^. | 9/11  QI met: A, B, C, D, E, F, G, H and I | 1. IG = CG 2. IG = CG |
| Ellman et al. (2009)  [2] | 77 (IG) + 180 (CG) third year students | 1. C: Preparatory session before clerkship. 2. W: Assignment: An end-of-life evaluation of a patient, personal interview of the patient, family member and caregivers and a written summary of assessment. 3. W: Case conference presentations: Reflections on student’s experience and group discussions.  - CG: Participation in preparatory session and case conference presentations but no assignment.   *🡪 Stages of cyclical process present: 1, 2, 3, 4 and 5* | 1. Once 30 minutes 2. 1 3. 8 monthly conferences (90 min.) | *CT*  Questionnaire (1 or 2 years after intervention): self-perceived preparedness in end-of-life care^A^. | 10/11  QI met: A, B, C, D, E, F, G, H, I and K | IG = CG but IG > CG for domains of interviewing/communicating and management of common symptoms |
| Elman et al. (2004)[3] | 51 (IG) + 59 (CG) third year students | 1. C: Interactive seminar about how to facilitate disclosure of family violence. Students practiced with SP. 2. W: Students got into contact with an unannounced SP in the clinical setting + received feedback.  - CG: Participation in interactive seminar but no contact with an unannounced SP in the clinical setting.   🡪 *Stages of cyclical process present: 1, 2, 3, 4, 5 (and 6?)* | 1. 2-hours once 2. 1 encounter with a SP | *CT*  Performing a consultation with SP (at the end of rotation)^D^. | 9/11  QI met: A, B, C, D, E, F, G, H and I | IG > CG for the frequency with which students inquired about family violence. |
| Aronoff et al. (2010)  [4] | 153 third year students | 1. C: Online modules about EBM + tasks + feedback from mentor. 2. W: Formulating clinical questions based on real patients + retrieving evidence + feedback.   *🡪 Stages of cyclical process present: 1, 2, 3, 4, 5 (and 6?)* | 1. 6 tasks in 18 weeks 2. 4 in 24 weeks | *Uncontrolled pre-& post-design*  Pre- and posttest of EBM skills^C^. | 9/11  QI met: A, B, C, D, E, F, G, H and I | Significant improvement in EBM skills. |
| **Reference** | **Participants** | **Intervention** | | **Study designs/ outcome measures** | **QI score** | **Study outcomes** |
|  |  | **Description** | **Duration / frequency** |  |  |  |
| Abu-Hijleh et al. (2005) [5] | 131 fifth and sixth year students | 1. C: Interactive anatomy sessions during surgical clerkship. 2. W: Surgical problem-solving sessions (based on real patients) during surgical clerkship: topic corresponding with anatomy sessions.   *🡪 Stages of cyclical process present: 1, 2, 3, 4 and 5* | 2 x 15 weekly sessions | *Uncontrolled pre-& post-design*  Pre- and post- K test^B^. | 10/11  QI met: A, B, C, D, E, F, G, H, I and K | Significant improvement in students' mean scores. |
| Mascola (2008)[6] | 13 students and 11 residents | 1. C: Meeting with mentor to elicit perceptions of the pros and cons of practicing EBM and to explore prior K, S and A. 2. C: Discussion sessions to convey how to apply EBM in a busy clinical practice. 3. W: Consideration of trainees’ concerns if discrepancies arose between trainee's decision and attending's preference during patient care. 4. W: Mentor modeled strategies to enhance self-efficacy in overcoming barriers to applied EBM practice.   *🡪 Stages of cyclical process present: 1, 2, 3, 4 and 5* | Six 10-15 minute discussion sessions over 6 months | *Pilot cohort study*   1. Attendings’ assessment of trainees' EBM S in managing actual patient scenarios^E^. 2. Pre- and posttest of K and S. | 10/11  QI met: A, B, C, D, E, F, G, H, I and K | 1. Majority of trainees demonstrated adequate proficiency of EBM S 2. Significant improvement in EBM K and S |
| Ellman et al. (2007) [7] | 121 third year students | 1. C: Preparatory session before clerkship. 2. W: Assignment: A comprehensive end-of-life evaluation of a patient, personal interview of the patient, family member and caregivers and a written summary of assessment. 3. W: Case conference presentations: Reflections on student’s experience and group discussions.   *🡪 Stages of cyclical process present: 1, 2, 3, 4, 5 (and 6?)* | 1. Onces 30 minutes 2. 1 3. 8 monthly conferences (90 min.) | *Qualitative study*  Students’ written reports^A^. | 10/11  QI met: A, B, C, D, E, F, G, H, I and J | Overall positive perceptions of the assignment and desire to broaden the experience to additional services. Many students stated that, had the assignment not been mandatory this learning would have been missed. |
| **Reference** | **Participants** | **Intervention** | | **Study designs/ outcome measures** | **QI score** | **Study outcomes** |
|  |  | **Description** | **Duration / frequency** |  |  |  |
| Haspel et al. (2012) [8] | 55 third year students | 1. C: Transition course about pathology. 2. W: Case conferences. 3. W: Pathology electives: facilitating understanding of the pathology of patient cases seen by students during the clerkship.   *🡪 Stages of cyclical process present: 1, 2, 3, 4 and 5* | 1. 60 min. 2. 90 min. twice a month 3. Monthly | *Evaluation study*  Pre- and post-questionnaire about ratings of the course^A^. | 9/11  QI met: B, C, D, E, F, G, H, I and J | No significant difference between the answers of the pre- and post-questionnaire. |
| Claxton et al. (2011) [9] | 41 (IG) + 41 (CG) internal medicine interns | ***Workplace to classroom interventions:***   1. W: Caring for real patients. 2. C: E-mails containing two Fast Facts and Concepts (FFAC) about palliative care topics: 1-page, practical, peer-reviewed, evidence-based summaries of key palliative care topics.  - CG: No FFAC e-mails were send.   *🡪 Stages of cyclical process present: 1* | 1 e-mail a week for 32 weeks | *RCT*  Pre- and post-:   1. K test^B^ (1 to 8 weeks after intervention). 2. Questionnaire: self-reported preparedness. 3. Questionnaire: satisfaction of the course. | 10/11  QI met: A, B, C, D, E, F, G, H, I and J | 1. IG > CG 2. IG > CG in 3 aspects of symptom management S 3. IG = CG |
| Davis et al. (2012)[10] | 42 students (year 2 or higher), 44 residents + 42 in surgical team (64 IG + 64 CG) | 1. W: Caring for real patients. 2. C: A video demonstrating proper chest tube placement (refreshment of prior K and S).  - CG: No video or any other instruction.   *🡪 Stages of cyclical process present: 1* | 3 minutes (once only) | *CT*  Placing a chest tube on TraumaMan task simulator^D^. | 10/11  QI met: A, B, C, D, E, F, G, H, I and J | IG > CG |
| Hirsh et al. (2012)[11] | 27 (IG) + 45 (CG) third year students | 1. W: Students follow patients through care pathways. 2. W: Rounds with Teaching Attending Physicians to review films and specimens of patients from the student cohorts. 3. C: Small group tutorials based on patient cases that best illustrated the weekly basic science topic + review K and S related to it. 4. C: Portfolio: notes of each patient encounter.  - CG: Students had a regular clerkship.   *🡪 Stages of cyclical process present: 1* | Weekly tutorials over a year | *CT*   1. OSCE^D^ 2. K Tests 3. Pre- and post-questionnaire about patient-centered beliefs. | 9/11  QI met: B, C, D, E, F, G, H, I and K | 1. IG > CG 2. IG = or > CG 3. IG > CG |
| **Reference** | **Participants** | **Intervention** | | **Study designs/ outcome measures** | **QI score** | **Study outcomes** |
|  |  | **Description** | **Duration / frequency** |  |  |  |
| Ogur et al. (2007)[12] | 18 (volunteers) + 8 (randomly selected) third year students | 1. W: Students follow patients through major care pathways. 2. W: Rounds with Teaching Attending Physicians to review films and specimens of patients from the student cohorts. 3. C: Small group tutorials based on patient cases that best illustrated the weekly basic science topic + review K and S related to it. 4. C: Portfolio: notes of each patient encounter.  - CG: Students had a regular clerkship.   *🡪 Stages of cyclical process present: 1* | Weekly tutorials over a year | *Post-intervention only*   1. Tests of K and S + OSCE^D^. 2. Clinical skills self-assessment. 3. Questionnaire about patient-centred attitudes. 4. Questionnaire + focus group about ratings of the course. 5. Patient logs. | 9/11  QI met: A, B, C, D, F, G, H, I and K | 1. IG = or > CG 2. IG > CG 3. IG = or > CG 4. Students in IG found the year more rewarding and felt better prepared 5. Students in IG logged equal or more exposure to all core diagnoses |
| Mainhard et al. (2004) [13] | 188 third and fourth year students | 1. W: Practice days: connected to the topics of the theoretical course and the skills programme. 2. W: Assignments for integration of K and S at W. 3. C: Coaching groups: Feedback on assignments + discussion and reflection with GP.   *🡪 Stages of cyclical process present: 1, 2, 3, 4, 5 (and 6?)* | 1. 8 days over 18 months 2. 60 3. Monthly | *Evaluation study*  Questionnaire about ratings of the course and elements contributing to the instructiveness of the programme^A^. | 7/11  QI met: A, B, C, F, G, H and J | Assignments, supervision in practice via coaching groups and by the GP had only a limited effect on the instructiveness as perceived by students. |
| Mauksch et al. (2013) [14] | 3 third year students and 19 fourth or fifth year students | 1. W: Paired observation: Two students (an observer and a ‘doctor’) see patients together. 2. C: Didactics: Mini-lectures on core communication topics connected to clinic days. 3. C: Review recorded patient consultations: Using observation form + discussing practical and theoretical issues with faculty.   *🡪 Stages of cyclical process present: 1, 2, 3, 4, 5 (and 6?)* | 1. During 3 weeks 2. Several sessions of 20-60 min. 3. Daily | *Evaluation study*   1. Pre- and post-questionnaire about self-confidence in communication S^A^. 2. Students’ and faculty’s ratings of the course. 3. Video analysis. | 8/11  QI met: B, C, E, F, G, H, I and K | 1. Noted increase in student self-confidence. 2. Students and faculty positive. 3. Videos showed frequency of S done by students |
| **Reference** | **Participants** | **Intervention** | | **Study designs/ outcome measures** | **QI score** | **Study outcomes** |
|  |  | **Description** | **Duration / frequency** |  |  |  |
| Jacobs et al. (2005)[15] | 37 residents and 54 fifth year students | 1. W: Ward-based training, group meetings and supervision from residents. 2. W: Logbook (to collect clinical experiences on which they can reflect on during meetings). 3. W: Halfway + final meetings for reflection and feedback. 4. C: Students return to medical school to reflect on their experiences and to prepare for the next clerkships.   *🡪 Stages of cyclical process present: 1, 2, 3, 4, 5 (and 6?)* | ND | *Evaluation study*  Questionnaire about ratings of the course^A^. | 9/11  QI met: A, B, C, D, F, G, H, I and K | Students appreciated the intervention as it offered a safe period to get used to their new role, to discuss experiences, individual goals and expectations and to reflect on their learning. |
|  |  | ***Interventions involving multiple connections between classroom and workplace:*** |  |  |  |  |
| Kerfoot et al. (2007) [16] | 133 third year students | 1. C: Web-based teaching programme on urology. 2. W: Urology rotation. 3. C: After completing the rotation, students received spaced educational e-mails (SEE) on urology. These had a short clinically relevant question or case scenario, followed by explanations of the answer, a summary of teaching points and a listing of 'take-home messages'.  - CG: Received SEE about two different topics.   *🡪 Stages of cyclical process present: 1, 2, 3, 4 and 5* | 22 weekly e-mails for a duration from 0 to 11 months | *RCT*   1. Pre- and post- K test^B^ (after web-based programme). 2. End-of-year K test (after SEE) 3. Self-reported SEE utilization patterns. | 11/11  QI met: All | 1. IG > CG for comparison post- and end-of-year test 2. IG > CG 3. IG > CG for reading the SEE |
| George et al. (2012) [17] | 26 second year residents | 1. C: One-on-one coaching sessions about EBM and information mastery skills. 2. W: Formulation of clinical questions departing from a real patient and searching for answers + feedback. 3. C: Formulation of personal learning goals, guided by coach.   *🡪 Stages of cyclical process present: 1, 2, 3, 4, 5 (and 6?)* | 5 monthly 1-hour meetings | *Uncontrolled pre-& post-design*   1. Pre- and post-K test. 2. Coach rated residents’ EBM S and K^C^. 3. Interview: Attitudes towards EBM sessions. | 8/11  QI met: B,C, E, F, G, H, I and J | 1. Significant improvement in total scores 2. Improvements from first to last session 3. Importance of one-on-one coaching |
| **Reference** | **Participants** | **Intervention** | | **Study designs/ outcome measures** | **QI score** | **Study outcomes** |
|  |  | **Description** | **Duration / frequency** |  |  |  |
| Macallan et al. (2009) [18] | 97 third year students | 1. W: Allocating case based on patient seen during clinical practice: Clinical PBL (CPBL). 2. C: Case presentation and identifying learning objectives. 3. W: Review at bedside. 4. C: Using textbook and online resources to answer learning objectives. 5. W: Summarising learning and revisit patient at bedside. 6. C: Case report.   *🡪 Stages of cyclical process present: 1, 2, 3, 4, 5 (and 6?)* | ND | *Evaluation study*   1. Questionnaire about ratings of the course. 2. Focus groups 3. Delphi consensus approach about components contributing to success of CPBL^A^. | 10/11  QI met: A, B, C, D, E, F, G, H, I and J | 1. Overall, students rated the course as positive. 2. Dominant theme was the value attached to contact with expert tutors. 3. Most statements referred to the CPBL process and structure. |
| Van Weel-Baumgarten et al. (2013) [19] | 673 students in year 4, 5 and 6 | 1. C: Preparation for clerkship: experiential sessions about communication with SPs and multisource feedback. 2. W: Practicing with real patients during the clerkship. 3. C: Reflection on clerkship in small groups and individual counseling sessions.   *🡪 Stages of cyclical process present: 1, 2, 3, 4, 5 (and 6?)* | 3 years | *Evaluation study*  Questionnaire about ratings of the course^A^. | 11/11  QI met: All | Students’ perceptions:  Good preparation for clerkship  Importance of communication taught integrated with medical content. |
| Bullock et al. (2013) [20] | 136 foundation year 1 and 2 trainees | 1. C: Induction training + shadowing prior to the workplace rotation. 2. W: Caring for real patients. 3. C: Access to medical texts, protocols and flow charts on a smartphone during rotation.   *🡪 Stages of cyclical process present: 1 and 2* | 1. 1 week 2. ND 3. unlimited | *Evaluation study*  Interviews with trainees and trainees’ narrative case reports rating of the course^A^. | 9/11  QI met: B, C, D, E, F, G, H, I and J | Both parts of intervention addressed different challenges in transition between C and W (e.g. role expectation, responsibilities). |

***Legend***

| C: Classroom  W: Workplace  K: Knowledge  S: Skills  ND: Not disclosed | IG: Intervention group  CG: Control group  RCT: Randomized controlled trial  CT: Controlled trial  SP: Simulation patient | ^A^ No level of Miller’s pyramid applicable  ^B^ “Knows” level of Miller’s pyramid  ^C^ “Knows how” level  ^D^ “Shows how” level  ^E^ “Does” level | - These refer to the highest outcome measurement of that study |
| --- | --- | --- | --- |

**Reference List**

1. Todsen T, Henriksen MV, Kromann CB, Konge L, Eldrup J, Ringsted C. Short- and long-term transfer of urethral catheterization skills from simulation training to performance on patients. BMC Med Educ. 2013;13:29.

2. Ellman MS, Rosenbaum JR, Cherlin E, Bia M. Effectiveness of an Integrated Ward-based Program in Preparing Medical Students to Care for Patients at the End of Life. American Journal of Hospice & Palliative Medicine. 2009;26(1):18-23.

3. Elman D, Hooks R, Tabak D, Regehr G, Freeman R. The effectiveness of unannounced standardised patients in the clinical setting as a teaching intervention. Med Educ. 2004;38(9):969-73.

4. Aronoff SC, Evans B, Fleece D, Lyons P, Kaplan L, Rojas R. Integrating Evidence Based Medicine Into Undergraduate Medical Education: Combining Online Instruction With Clinical Clerkships. Teach Learn Med. 2010;22(3):219-23.

5. Abu-Hijleh MF, Chakravarty M, Al-Shboul Q, Kassab S, Hamdy H. Integrating applied anatomy in surgical clerkship in a problem-based learning curriculum. Surg Radiol Anat. 2005;27(2):152-7.

6. Mascola AJ. Guided mentorship in evidence-based medicine for psychiatry: A pilot cohort study supporting a promising method of real-time clinical instruction. Acad Psychiatry. 2008;32(6):475-83.

7. Ellman MS, Rosenbaum JR, Bia M. Development and implementation of an innovative ward-based program to help medical students acquire end-of-life care experience. Acad Med. 2007;82(7):723-7.

8. Haspel RL, Bhargava P, Gilmore H, Kane S, Powers A, Sepehr A, et al. Successful implementation of a longitudinal, integrated pathology curriculum during the third year of medical school. Arch Pathol Lab Med. 2012;136(11):1430-6.

9. Claxton R, Marks S, Buranosky R, Rosielle D, Arnold RM. The educational impact of weekly e-mailed fast facts and concepts. J Palliat Med. 2011;14(4):475-81.

10. Davis JS, Garcia GD, Wyckoff MM, Alsafran S, Graygo JM, Withum KF, et al. Use of mobile learning module improves skills in chest tube insertion. J Surg Res. 2012;177(1):21-6.

11. Hirsh D, Gaufberg E, Ogur B, Cohen P, Krupat E, Cox M, et al. Educational Outcomes of the Harvard Medical School-Cambridge Integrated Clerkship: A Way Forward for Medical Education. Acad Med. 2012;87(5):643-50.

12. Ogur B, Hirsh D, Krupat E, Bor D. The Harvard Medical School-Cambridge Integrated Clerkship: An innovative model of clinical education. Acad Med. 2007;82(4):397-404.

13. Mainhard MT, van den Hurk MM, Van de Wiel MWJ, Crebolder HFM, Scherpbier AJJA. Learning in a clinical education programme in primary care: the Maastricht Adoption Programme. Med Educ. 2004;38(12):1236-43.

14. Mauksch L, Farber S, Greer HT. Design, Dissemination, and Evaluation of an Advanced Communication Elective at Seven US Medical Schools. Acad Med. 2013;88(6):843-51.

15. Jacobs J, Salas A, Cameron T, Naguwa G, Kasuya R. Implementing an online curriculum management database in a problem-based learning curriculum. Acad Med. 2005;80(9):840-6.

16. Kerfoot BP, Dewolf WC, Masser BA, Church PA, Federman DD. Spaced education improves the retention of clinical knowledge by medical students: a randomised controlled trial. Med Educ. 2007;41(1):23-31.

17. George P, Reis S, Nothnagle M. Using a Learning Coach to Teach Residents Evidence-based Medicine. Fam Med. 2012;44(5):351-5.

18. Macallan DC, Kent A, Holmes SC, Farmer EA, McCrorie P. A model of clinical problem-based learning for clinical attachments in medicine. Med Educ. 2009;43(8):799-807.

19. van Weel-Baumgarten E, Bolhuis S, Rosenbaum M, Silverman J. Bridging the gap: How is integrating communication skills with medical content throughout the curriculum valued by students? Patient Educ Couns. 2013;90(2):177-83.

20. Bullock A, Fox F, Barnes R, Doran N, Hardyman W, Moss D, et al. Transitions in medicine: Trainee doctor stress and support mechanisms. Journal of Workplace Learning. 2013;6:368-82.
